# Unexpected SARS-CoV-2 cardiorespiratory arrest in a myopathy patient undergoing immunosuppressive treatment: A case report

**DOI:** 10.1097/MD.0000000000021377

**Published:** 2020-07-24

**Authors:** Nicholas L. DePace, Stephen Soloway, David Roshal, Alyxandra Morgan Soloway, Joe Colombo

**Affiliations:** a Pennsylvania Hospital of the University of Pennsylvania Health System, Philadelphia, PA; b Stephen Soloway, Rheumatology, Vineland, NJ, USA; c Jefferson Health, New Jersey Division, Rowan University School of Osteopathic Medicine, Stratford, NJ; d Franklin Cardiovascular Associates, PA, Sewell, NJ.

**Keywords:** cardio-respiratory arrest, coronavirus (COVID-19), dysphagia, immunosuppressive therapy, inflammatory myopathy

## Abstract

**Rationale:**

It is recommended that patients with Rheumatic diseases that are at high risk of developing active infections be screened for Tuberculosis, Hepatitis B, and Hepatitis C before receiving second-line immunosuppressive therapies. With the emergence 2019 novel coronavirus (SARS-CoV-2), expanded guidelines have not been proposed for screening in these patients before starting advanced therapy.

**Patient concerns:**

We present an unique circumstance whereas a patient with a 5 year history of inflammatory muscle disease, diagnosed by clinical history and muscle biopsy with elevated creatine kinase levels, suffered a hypoxemic cardiopulmonary arrest due to asymptomatic SARS-CoV-2 after receiving advanced immunosuppressive therapy.

**Diagnoses:**

The patient presented with an acute exacerbation of inflammatory muscle disease with dysphagia, muscle weakness, and elevated creatine kinase.

**Interventions:**

After no improvement with intravenous immunoglobulin the patient received mycophenolate and plasma exchange therapy.

**Outcomes:**

Subsequently the patient suffered a fatal hypoxemic cardiopulmonary arrest. Polymerase chain reaction test was positive for SARS-CoV-2 RNA.

**Lessons:**

We conclude that rheumatic patients, asymptomatic for SARS-CoV-2 infection, be screened and tested before initiating second-line immunosuppressive treatment.

## 1 Introduction

Inflammatory myopathies represent a heterogeneous group of disorders which include dermatomyositis, polymyositis, immune-mediated necrotizing myopathy (IMNM), and inclusion body myositis and may occur in both children and adults.^[1]^ There are various strategies for treating inflammatory myopathies especially IMNM. IMNM accounts for approximately one-fifth of all inflammatory myopathies and present with severe muscle weakness and high creatinine levels. They are often seen after viral infections, malignancies (cancer) or connective tissue disorder such as rheumatoid arthritis, lupus, or scleroderma, and can be seen in patients taking statins.^[1]^

Many of these patients have resistance to conventional immunosuppressive therapy.^[2]^ IMNM is distinguished by the absence of primary inflammation on muscle biopsy and may be associated with myositis-specific autoantibodies. Prompt treatment is important especially in patients who develop acute or progressive swallowing or breathing abnormalities from difficulty with skeletal muscle function. Prednisone is a first-line treatment, but is oftentimes ineffective and second-line treatment needs to be employed, which may include disease-modifying agents, such as Methotrexate, Azathioprine or Mycophenolate Mofetil. Additional second-line treatment includes intravenous immunoglobulin (IVIG). Recent research has suggested a high rate of response to Rituximab in patients with autoimmune myopathies.^[3]^

Immunosuppressive therapy increases the risk of infection including aspiration pneumonia.^[3,4]^ Pneumococcal vaccine and yearly Influenza vaccinations are recommended. Before starting second-line treatment, it has also been recommended to screen for tuberculosis and hepatitis B and C. There are no consensus guidelines for pneumocystis pneumonia. With the emergence and spread of the 2019 novel coronavirus (COVID-19^[5]^ or SARS-CoV-2), a disease that also affects the respiratory system, it has become imperative to consider this virus when beginning patients on immunosuppressive therapies. Patients infected with COVID-19 present with fever, cough, sore throat, breathlessness, fatigue, malaise, and other symptoms. This is predominately an upper respiratory infection. However, a large subset of patients may be asymptomatic.^[6]^ Written informed consent was obtained from the patient's family for publication of this case report.

We described a case of IMNM on escalating immunosuppressive therapy. The patient developed sudden cardiopulmonary arrest due to unsuspected COVID-19. We propose new guidelines to follow for patients for whom initiating or escalating immunosuppressive therapy is being considered, especially if they have comorbidities.

## 2 Case history

A 50-year-old African-born male was admitted to the Jefferson New Jersey Division Hospital from a rehabilitation facility with progressive Dysphagia and Pulmonary Aspiration. The patient had a 5-year history of diagnosed IMNM, from which he initially responded to IVIG, despite becoming hypertensive with the IVIG treatment. He was diagnosed with the exclusion of the following antibodies: he was found negative for 3-hydroxy-3-methyl-glutaryl-coenzyme A antibodies, anti-signal recognition particle antibodies, anti-myositis specific autoantibody and other Synthetase antibodies, as well as anti-melanoma differentiation-associated gene 5-antibody. The diagnosis was also based on muscle biopsies. He had multiple muscle biopsies which showed positive indications for non-specific Myositis (consistent with necrotizing myopathy). He had chronic muscle weakness and had frequent flare-ups. At times, he had creatine kinase (CK) elevations over 30,000 IU/L (normal CK: 40–172 IU/L). The patient had 2 brief hospitalizations at a nearby community hospital and had received intravenous Solu-Medrol (Methylprednisolone) for 3 days. He was started on 150 mg/d, Azathioprine in addition to pre-existing 20 mg once a week, methotrexate. He was on high dose oral Prednisone. He was transferred to subacute rehab from his second hospitalization and began developing severe dysphagia, swallowing problems, and could not eat. He was promptly transferred to the emergency room of a different hospital with a CK elevation of 2343 IU/L.

At the time of the admission to this emergency room, the patient appeared in no acute distress. He had past medical history of hypertension and type 2 diabetes. He did not smoke or drink alcohol, had recent, mild weight loss. Physical exam was noted for decrease muscle strength in all proximal muscles (grade 2/5) and bilateral mild edema. He had Rhonchi over the lung fields, and his blood pressure (BP) was 141/79 mm Hg, heart rate was 84 beats per minute (bpm), Temperature was 98.6°, Respirations were 18 bpm, and pulse oxygenation was 98%. The patient complained of a mild cough from aspiration and dysphagia. The patient also had weakness and fatigue. Chest X-ray showed bibasilar atelectasis. Aspiration could not be excluded and he was started on IV Unasyn. On admission, computed tomography of the chest also showed bibasilar atelectasis and no pulmonary infiltrate. White blood count was 6.2/mL (normal: 3.7–10.5/mL). Electrocardiogram showed normal sinus rhythm, and nonspecific ST-T wave changes. Troponin level was normal.

The patient was admitted with a diagnosis of progressive Dysphagia with IMNM not responsive to high-dose oral steroids. The patient was evaluated by a Gastroenterology specialist who diagnosed Dysphagia to solids and ordered a Dobhoff to be initiated for 2 feedings. The patient had an abnormal video swallow and was seen by Speech Therapy. He was diagnosed with mild oral and severe Pharyngeal Dysphagia. Inflammation markers, including C-reactive protein, were not elevated. Procalcitonin was normal.

The patient underwent an empiric treatment of 1 g/kg IVIG for 5 days without improvement. He was also started on 500 mg q 12 hours, CellCept (Mycophenolate). The patient after receiving IVIG intravenous fluids went into mild Heart Failure and became hypoxic, that was reversed with diuretic therapy. He did become Hypertensive with IVIG treatment, similar to his past history with this agent. Echocardiogram showed normal left ventricular systolic function and mild diastolic dysfunction.

The patient had a brief stay in the intensive care unit for what appeared to be an exacerbation of chronic hypercapnic respiratory failure due to underlying neuromuscular weakness complicated by volume overload which responded to treatment. Neuromuscular weakness (including swallowing weakness) and being bedridden can lead to diaphragm weakness that can lead to Dysphagia. The dysphagia did not improve. He did have impaired vital capacity and negative inspiratory pressure was documented. Communication was established with the patient's outpatient rheumatologist almost on a daily basis.

The patient underwent chest physiotherapy with Albuterol and Mucomyst treatments. The patient was transferred to the telemetry floor and remained stable on room air with normal pulse oxygenation. He had no respiratory distress or audible secretions detected. After the patient did not show improvement with IVIG, an outside Rheumatology consultation recommended Plasma Exchange therapy (PLEX). A vascular access was placed and the patient underwent 1 treatment and appeared to be quite stable. The patient was put on IV methylprednisolone 1000 mg every 24 hours for 5 consecutive days. His CK values dropped from 2,343 IU/L to 1588 IU/L to 1446 IU/L and then to 690 IU/L (normal: 40–172 IU/L). He stated that he could swallow somewhat better, but was still receiving Dobhoff feedings. He was Euvolemic, clinically, and not in heart failure.

On the patient's tenth hospital day, before the second PLEX, the patient had a brief episode of desaturation; down to 90% oxygen saturation. He received 2 L of oxygen by nasal cannula and became hypertensive with a BP of 202/101 mm Hg. He then received 20 mg of intravenous Furosemide. A chest X-ray showed no new abnormalities. With the low-dose oxygen, the patient stabilized with a Pulse-Ox of 95%, BP of 100/66 mm Hg, Temp. of 98°, and respirations of 18 bpm, and comfortable.

On the eleventh hospital day the patient became acutely hypoxic and rapid response team was called. His BP dropped to 69/39 mm Hg. Respirations rose to 24 bpm, Temp. was 97.4°, and he was placed on high flow oxygen at 100%. His white blood cell count was 4.9/mL (normal: 3.7–10.5/mL), Hemoglobin was 13.9 g/dL (normal: 12.8–16.3 g/dL), Troponin was 0.08 ng/mL (normal: <0.02 ng/mL), blood sugar was 176 mg/dL (normal: 70–105 mg/dL), brain natriuretic peptide was normal at 52 pg/mL (normal: 0–100 pg/mL), and pulse-ox was 89%. The patient became lethargic and was placed on non-breather. He was then intubated. He was put on Levophed to main a mean arterial pressure greater than 65 mm Hg. He subsequently lost pulses and advanced cardiovascular life support was instituted. He had return to spontaneous pulses; however, the intensive care unit team transitioned the patient to comfort care after discussion with family members. The patient expired shortly thereafter. The patient had a nasal pharyngeal swab taken to test for COVID-19 (SARS-CoV-2) by polymerase chain reaction assay for SARS-CoV-2 RNA. Two days later, it was disclosed the patient tested positive for COVID-19.

## 3 Discussion

This is a case of a patient with a 5-year history of IMNM who previously responded to IVIG, but with the most recent acute flare-up did not have a good response. Despite high-dose intravenous steroids (IVIG), mycophenolate (CellCept), and subsequent PLEX, the patient did not respond and had an acute deterioration with Hypoxemia, Hypotension, and Cardiorespiratory Arrest, all of which occurred suddenly. Polymerase chain reaction assay of SARS-CoV-2 RNA disclosed COVID-19. Pre-mortem, before the patient's cardiopulmonary arrest, this infection was not suspected. The patient was Euvolemic and had no fever or cough immediately before sudden cardiopulmonary arrest. He had no significant radiographic infiltrates and no signs of elevated inflammatory markers. The patient was, however, on high-dose steroids which may have suppressed fever and the inflammatory response. He also had multiple co-morbidities, including hypertension and type 2 diabetes, which increased his risk of an adverse outcome with exposure to COVID-19.

Among subsets of patients at high risk of developing severe infections are patients with Rheumatic diseases including lupus, rheumatoid arthritis, scleroderma, inflammatory myopathies, and vasculitis.^[3,7]^ The European League Against Rheumatism released guidance for patients with rheumatic and musculoskeletal diseases receiving immunosuppressive therapy, including biological agents and disease-modifying anti-rheumatic drugs.^[8]^ In addition to recommendations for screening for tuberculosis and hepatitis B and C, we propose that this subset of patients should receive screening for COVID-19 before initiating or escalating Immunosuppressive therapy.

**Figure 1:**
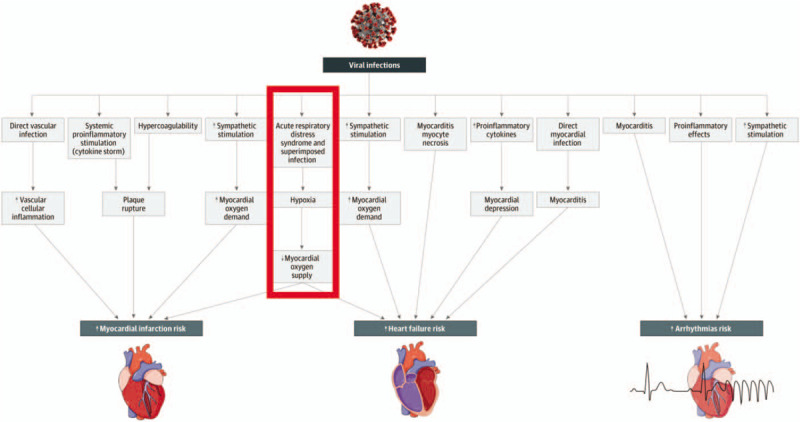
A recent JAMA Cardiology article published the above chart indicating the potential mechanisms for acute effects of viral infections on the cardiovascular system [9, JAMA Cardiol. Published online March 27, 2020. doi:10.1001/jamacardio.2020.1286]. The pathway outlined in red represents the patient in this case study.

Coronavirus can cause viral pneumonia. This patient showed no evidence of radiographic viral pneumonia or increased biomarkers. In addition, COVID-19 can cause myocardial damage and myocarditis. This patient's echocardiogram showed no evidence of myocardial impairment and Troponins and brain natriuretic peptide were negative for myocardial injury during the patient's hospital course. Also, acute viral infections can be responsible for acute coronary syndrome, and plaque rupture can trigger and precipitate Acute Coronary Syndromes, but this was not demonstrated in this case; although hypoxemic respiratory failure and inflammation may precipitate arrhythmias and depression of myocardial function (see Fig. 1).^[9]^ It appears that the patient developed an abrupt, overwhelming, acute Respiratory Distress Syndrome, which came on precipitously as a result of COVID-19 in a very immunocompromised host with multiple co-morbidities.

## 4 Conclusions

We believe this to be an instructive case and we discuss recommendations for expanded guidelines on using Immunosuppressive therapy in subsets of patients with high risk rheumatological diseases. To this end, we present a patient with IMNM who required aggressive Immunosuppressive therapy because of acute relapse and significant progression of dysphagia. Unexpectedly, the patient had an acute hypoxemic episode and fatal cardiopulmonary arrest. He had no significant symptoms or radiographic findings consistent with COVID-19 acute infection. We propose that Rheumatological patients, even when asymptomatic, be tested for COVID-19 before initiating second-line Immunosuppressive therapy treatment, especially if they have other co-morbidities.

## Author contributions

**Conceptualization:** Nicholas L. DePace, Stephen Soloway, David Roshal.

**Formal analysis:** Nicholas L. DePace, Stephen Soloway, David Roshal, Joe Colombo.

**Investigation:** Nicholas L. DePace, Stephen Soloway, David Roshal, Joe Colombo.

**Project administration:** Nicholas L. DePace.

**Resources:** Alyxandra Morgan Soloway.

**Review:** Alyxandra Morgan Soloway.

**Supervision:** Joe Colombo.

**Visualization:** Joe Colombo.

**Writing:** Joe Colombo.
